# Evaluating the Immunogenicity of an Intranasal Microparticle Combination Vaccine for COVID-19 and Influenza

**DOI:** 10.3390/vaccines13030282

**Published:** 2025-03-07

**Authors:** Sharon Vijayanand, Smital Patil, Priyal Bagwe, Revanth Singh, Emmanuel Adediran, Martin J. D’Souza

**Affiliations:** Vaccine Nanotechnology Laboratory, Center for Drug Delivery and Research, College of Pharmacy, Mercer University, Atlanta, GA 30341, USA; sharon.c.p.vijayanand@live.mercer.edu (S.V.); smitalrajan.patil@live.mercer.edu (S.P.); priyal.bagwe@live.mercer.edu (P.B.); revanth.singh.sateesh@live.mercer.edu (R.S.); emmanuel.adediran@live.mercer.edu (E.A.)

**Keywords:** intranasal vaccine, infectious diseases, microparticles, immunogenicity, combination vaccine, COVID-19, influenza, antibody levels, cellular responses

## Abstract

Background: Infectious respiratory pathogens like SARS-CoV-2 and influenza frequently mutate, leading to the emergence of variants. This necessitates continuous updates to FDA-approved vaccines with booster shots targeting the circulating variants. Vaccine hesitancy and needle injections create inconvenience and contribute to reduced global vaccination rates. To address the burden of frequent painful injections, this manuscript explores the potential of non-invasive intranasal (IN) vaccine administration as an effective alternative to intramuscular (IM) shots. Further, as a proof-of-concept, an inactivated combination vaccine for COVID-19 and influenza was tested to eliminate the need for separate vaccinations. Methods: The methods involved encapsulating antigens and adjuvants in poly(lactic-co-glycolic acid) (PLGA) polymer matrices, achieving over 85% entrapment. The vaccine was evaluated in vitro for cytotoxicity and immunogenicity before being administered to 6–8-week-old Swiss Webster mice at weeks 0, 3, and 6. The mice were then assessed for antibody levels and cellular responses. Results: The intranasal microparticle (IN-MP) vaccine induced an innate immune response, autophagy, and were non-cytotoxic in vitro. In vivo, the vaccine led to high levels of virus-specific serum IgM, IgG, and IgA binding antibodies, as well as elevated IgG and IgA levels in the lung wash samples. The antibodies generated demonstrated neutralizing activity against the SARS-CoV-2 pseudovirus. Furthermore, the IN-MP vaccine prompted increased antigen-specific CD4^+^ and CD8^+^ T-cell responses in the vaccinated mice. Conclusions: The IN-MP combination vaccine produced immune responses comparable to or higher than the IM route, indicating its potential as an alternative to IM injections.

## 1. Introduction

Infectious respiratory pathogens like SARS-CoV-2 and influenza have the capacity to frequently mutate into variant strains due to antigenic drift [1,2]. Antigenic drift occurs when small mutations in the genetic make-up of the virus lead to slight modifications in the antigenic structure of the original pathogen [1,3]. As a result, vaccines manufactured based on the original structure of the pathogen result in reduced efficacy against the mutated variants [4]. Regulatory agencies convene annually to discuss and recommend influenza virus strains for inclusion in the seasonal flu vaccine. For the 2024–2025 flu season, the FDA has recommended transitioning from a quadrivalent to a trivalent vaccine, removing the influenza B/Yamagata Lineage that is no longer a threat, and including two influenza A viruses (H1N1 and H3N2) and one influenza B virus [5]. Since the COVID-19 pandemic, the agency has approved and authorized updated mRNA COVID-19 vaccines which better protect against the circulating strains of SARS-CoV-2. On August 2024, the FDA granted emergency use authorization for the updated mRNA COVID-19 vaccine which includes a monovalent component corresponding to the Omicron variant KP.2 strain of SARS-CoV-2 [6]. The increased frequency of immunizations for multiple pathogens may lead to reduced vaccination rates due to the inconvenience and discomfort of receiving multiple vaccines and needle injections [7,8]. This places additional burden on healthcare professionals who are required to administer the vaccines, especially during a pandemic [9]. Consequently, there is a growing need for a more patient-friendly vaccination system that maintains efficacy while being pain-free and efficient.

The IN route of vaccine administration is a non-invasive method and is considered a highly desirable strategy for protecting against infectious diseases such as SARS-CoV-2 and influenza, which enter through the mucosal sites [10]. This is because IN vaccination can induce robust systemic as well as mucosal immune responses, which can fight the pathogen at the entry site, preventing viral replication and shedding [11]. The nasal mucosa is lined with nasal-associated lymphatic tissue (NALT) and pathogens cleared through NALT can generate both systemic and mucosal immune responses [12]. An important component of mucosal immunity is secretory IgA (SIgA) [13]. SIgA, secreted at a higher percentage in the mucosal sites in its dimeric form, plays a vital role in defending the invading pathogens at the point of entry [13,14]. In addition to it superiority in immune response generation against infectious pathogens, the IN route can also be a cost-effective and efficient alternative to IM injections, especially in developing countries [11]. However, a key challenge in developing a safe and efficacious IN vaccine is the selection of antigens and adjuvants [11]. Here, we explored the use of inactivated vaccine antigens and a combination of adjuvants to potentiate the immune response.

An inactivated form of the virus maintains the original structure of the virus, including the antigenic epitopes necessary for triggering an immune response [15]. When an inactivated virus is introduced to the host immune system, it is recognized as an extracellular antigen and is taken up by antigen presenting cells (APCs), processed and presented through the MHC II pathway, activating a CD4^+^ helper T-cell response and stimulating antibody production [16]. It is important to note that a CD8^+^ cytotoxic T-cell response can lead to the destruction of infected cells, making it a desirable component for viral vaccines [17]. In an effort to elicit both CD4^+^ and CD8^+^ T-cell responses, the antigens were encapsulated in a polymeric matrix using PLGA. Polymeric microparticles, being larger in size (1–3 μm), are easily recognized by antigen presenting cells and phagocytosed [18]. Additionally, these microparticles have the capability to induce autophagy, which has been linked to antigen presentation [19]. Autophagy is also investigated for its role in facilitating antigen cross presentation of extracellular antigens via the MHC-I pathway, leading to an enhanced CD8^+^ T-cell-mediated response and improved viral clearance [20]. Furthermore, the polymer encapsulation serves to protect the vaccine cargo from degradation by enzymes in tissue fluids [21]. It is also worth noting that particle-based vaccines have demonstrated stability at room temperature, thus reducing the need for refrigerated storage conditions [21].

In this study, the potential of using a combination of Alhydrogel^®^ (Alum) (InvivoGen, San Diego, USA), AddaVax™ (MF59-like) (InvivoGen, San Diego, USA), and CPG 7909 (BEI Resources, Manassas, VA, USA) as adjuvants for an inactivated combination vaccine is explored. Previously, the adjuvants Alhydrogel^®^ and AddaVax™ were tested in an inactivated SARS-CoV-2 MP vaccine, and the resulting immune responses were evaluated in a preclinical vaccine study and reported [22]. Alum, known for eliciting a Th2-biased response, and MF59, recognized for inducing a mixed Th1/Th2 response, were found to produce high levels of antibody titers and, to some extent, cellular responses when used in combination [23,24]. On the other hand, CPG 7909, is a TLR9 agonist oligodeoxynucleotide, known to act as a potent enhancer of Th-1 like immune responses, capable of inducing significant cellular responses, thereby contributing to an overall robust immune response [25,26]. Consequently, in this study, Alhydrogel^®^, Adda-Vax™, and CPG 7909 were utilized together as adjuvants to augment the immunogenicity of the inactivated antigens.

The main objective of this study was to assess a prototype IN combination vaccine for SARS-CoV-2 and influenza. The vaccine utilized inactivated viral antigens enclosed in polymer matrices along with adjuvants. Initially, the vaccine’s potential cytotoxicity and innate immune response induction were examined in vitro. Subsequently, the vaccine was tested in mice to evaluate its ability to elicit specific antibody levels and cellular responses. Additionally, the intranasal microparticle (IN-MP) vaccine was compared to the intramuscular (IM) vaccine suspension to analyze the differences in immune responses between the two routes of administration and the particulate versus suspension form of the antigen. The study’s findings provide support for the use of polymeric microparticles for vaccine delivery and the IN route of administration as an alternative to IM injections.

## 2. Materials and Methods

### 2.1. Materials

Poly (lactic-co-glycolic acid) (75:25) was procured from Evonik Industries (Essen, Germany). Polyvinyl alcohol (PVA) (Avg Mol Wt. 30,000–70,000), dichloromethane (DCM), Trehalose dihydrate, and Lipopolysaccharides (LPSs) from Escherichia coli O111:B4 were purchased from Sigma-Aldrich (St. Louis, MO, USA). The CYTO-ID^®^ autophagy detection kit was obtained from Enzo Life Sciences (Farmingdale, NY, USA). The following reagents were obtained through BEI Resources (Manassas, VA, USA), NIAID, NIH (Bethesda, MD, USA): SARS-Related Coronavirus 2, Isolate USA-WA1/2020, Heat Inactivated, NR-52286; Influenza A Virus, A/California/04/2009 (H1N1)pdm09, BPL-Inactivated, NR-49450; SARS-Related Coronavirus 2, Wuhan-Hu-1 Spike D614G-Pseudotyped Lentivirus, Luc2/ZsGreen, NR-53819; Human Embryonic Kidney Cells (HEK-293T) Expressing Human Angiotensin-Converting Enzyme 2, HEK-293T-hACE2 Cell Line, NR-52511; CpG 7909 Adjuvant, NR-52393; Influenza A virus, A/Puerto Rico/8-WG/1934 (H1N1), NR-29029. AddaVax™ (MF59) and Alhydrogel^®^ and were purchased from InvivoGen (San Diego, CA, USA). Fetal bovine serum (FBS), Dulbecco’s Modified Eagle’s Medium (DMEM, Non-essential amino acids, and penicillin/streptomycin were procured from American Type Culture Collection (ATCC) (Manassas, VA, USA). Murine dendritic cells (DCs) were a gift from Kenneth L. Rock at the Dana-Farber Cancer Institute, Inc., Boston, MA, USA). Six–eight-week-old Swiss Webster mice were procured from Charles River Laboratories (Wilmington, MA, USA). HRP-tagged secondary goat anti-mouse antibodies IgM, IgG, and IgA were procured from Invitrogen (Rockford, IL, USA). MTT (3-(4,5-Dimethylthiazol-2-yl)-2,5-Diphenyltetrazolium Bromide), Allophycocyanin (APC)-labeled anti-mouse CD4 and fluorescein isothiocyanate (FITC)-labeled anti-mouse CD8 antibodies were obtained from Invitrogen™, Thermofisher Scientific (Waltham, MA, USA). Luciferase assay system was purchased from Promega (Madison, WI, USA). Polybrene transfection reagent was purchased from Millipore sigma (St. Louis, MO, USA).

### 2.2. Formulation and Characterization of Antigen/Adjuvant Microparticles

Heat-inactivated SARS-CoV-2 and BPL-inactivated Influenza A H1N1 virus were utilized as model antigens for the purpose of establishing a proof-of-concept. The antigen MP and adjuvant MP (Alhydrogel^®^/AddaVax™/CPG 7909) were prepared using a double emulsion method with solvent evaporation, as previously described [27]. To achieve this, a 2% *w*/*v* solution of PLGA in DCM served as the organic phase and the antigen/adjuvant in a phosphate buffer (pH 7.4, aqueous phase) was added to the organic phase and emulsified for 2 min (30 s on/30 s off) using a probe homogenizer at 17,000 RPM. The primary emulsion containing the antigen/adjuvant in organic phase (primary emulsion) was added to a secondary aqueous phase containing PVA in deionized water (0.1% *w*/*v*) for emulsified 2 min using a probe homogenizer at 17,000 RPM to form a double emulsion. The final emulsion was kept stirring at 500 RPM for 5 h to remove the residual DCM via solvent evaporation. The excess PVA was removed by washing with deionized water followed by centrifugation at 17,000 RPM for 10 min. The MP was resuspended with 1 mL of 2% *w*/*v* of trehalose in deionized water to serve as a cryoprotectant. The MP formulations were freeze-dried to obtain the dry product. The percent recovery yield of the lyophilized product, size and size distribution, and surface charge of the MP was measured as described previously [22]. The percent encapsulation efficiency (%EE) of the antigen was measured using an indirect method [21]. Briefly, 5 mg of the antigen-loaded MP was weighed and digested with 1 mL of DCM to dissolve the PLGA matrix at room temperature. The protein antigen was extracted by centrifugation and solvent evaporation and the protein concentration was determined using a micro-Bicinchoninic acid (BCA) assay as per the manufacturer’s instructions. The % EE was calculated using a previously described formula [21].

### 2.3. Assesing Nitric Oxide (NO) Production by Stimulated DCs

DCs, are APCs that release various chemical entities when exposed to external stimuli, which in turn activate the downstream signaling pathways involved in generating an immune response [28]. One of the enzymes produced by dendritic cells is Inducible Nitric Oxide Synthase (iNOS), which plays a key role in generating significant amounts of NO that helps in combating invading pathogens as a critical component of the innate immune response [29]. In this study, the release of nitrite, a stored form of NO, by dendritic cells upon stimulation with MP was assessed using Griess’ nitrite assay, using a previously established method [22]. To conduct the assay, murine DCs were plated in a 96-well plate at a density of 1 × 10^4^ cells/well and then pulsed with a calculated amount of antigen MP and adjuvant MP, followed by an incubation period of 24 h at 37 °C. The different groups tested and their corresponding dose/well are listed and described in Table 1. The dose of the antigen/adjuvant used for the in vitro studies was 1/10th of the dose used for the in vivo testing. The dose was selected based on the previous work performed in the lab and published literature and is calculated based on the antigen/adjuvant concentration entrapped in the MP matrix (loading 1% *w*/*w*) [27]. After 24 h, the supernatants (50 μL/well) were transferred to a fresh 96-well plate, and a series of steps were followed to assess the release of nitrite using Griess’ nitrite assay. This involved the addition of sulfanilamide (1%) in phosphoric acid (5%) to each well, followed by an incubation period of 5–10 min at room temperature, protected from light. Subsequently, 50 μL of NED (0.1%) (N-1-naphthyl ethylenediamine dihydrochloride) solution in deionized water was added to each well and incubated at room temperature for 5–10 min, also protected from light. The appearance of a purple/magenta color indicated the release of NO, and the absorbance at 540 nm was measured using a plate reader (Bio Tek Synergy, BIO-TEK Instruments, Winooski, VT, USA). Finally, a sodium nitrite standard curve was used to quantify the nitrite content released by the DCs.

### 2.4. In Vitro Cytotoxicity Assessment of Vaccine and Adjuvant Microparticles

Previously, the cytotoxicity of the inactivated SARS-CoV-2 MP (iSCoV2 MP), Alhydrogel^®^ MP, and Adda-Vax™ MP was assessed and reported [27]. Here, the cytotoxicity of the inactivated Influenza virus MP (IIV MP) and CPG 7909 MP were assessed as described previously [27,30]. Briefly, DCs were plated at a cell density 1 × 10^4^ cells/well in a 96-well plate. Then, two-fold serial dilutions of the antigen/adjuvant MP (31.25 µg/mL to 125 µg/mL) were prepared in complete DMEM (cDMEM; DMEM high glucose medium with 2 mM L-glut, 1% Penicillin-streptomycin, sodium pyruvate, 10% FBS). The diluted MP suspensions were added in triplicates to each well and incubated for 24 h at 37 °C. Following incubation, the media was aspirated, and the cells were washed with PBS. 10 μL/well of MTT reagent was added first, and 90 μL/well of cDMEM was added to make up the volume to 100 μL/well. The cells with the MTT reagent were incubated for 4 h at 37 °C, protected from light. After 4 h, 100 μL/well of DMSO was added to dissolve the purple formazan crystals formed as function of metabolically active cells. The absorbance was measured at 570 nm using a plate reader. Cells that did not receive any treatment were used as the positive control, while cells that were treated with 50 μL/well (25% *v*/*v*) of DMSO served as the negative control.

### 2.5. Autophagy

The role of autophagy in regulating DC functions such as maturation, TLR stimulation, cytokine production, antigen presentation and subsequent T-cell activation has previously been evaluated and reported [19,20,31,32]. Autophagy is linked to antigen presentation via the MHC class II molecules and antigen cross presentation via the MHC class I pathway [19]. Here, the formation of autophagosomes in DCs upon exposure to different treatment groups was assessed using a CYTO-ID^®^ autophagy detection kit (Enzo Life Sciences, Farmingdale, NY, USA) as per the manufacturers protocol. The quantitative analysis was performed using flow cytometry and qualitative images were obtained using fluorescent live cell imaging technique. First, DCs were plated in a 24-well plate at a seeding density of 5 × 10^4^ cells. At 70% confluency (after 24 h), the cells were treated with the following: Cells only, (iSCoV-2 + IIV) suspension, (iSCoV-2 + IIV + adjuvants) suspension, (iSCoV-2 + IIV) MP, (iSCoV-2 + IIV + adjuvants) MP. The dose of each group is the same as the dose specified in Table 1 for the respective groups. The treated cells were incubated for 24 h at 37 °C and 5% CO_2_. For fluorescence microscopy, cells were washed with phosphate-buffered saline (PBS), pH 7.4, and stained with CYTO-ID^®^ dye and Hoechst 33342 Nuclear stain for 30 min at 37 °C. After the incubation period, the unbound dye was washed, and the cells were observed under DAPI and FITC filters of a fluorescence microscope (Lionheart FX, Biotek, VT, USA). The cell nucleus was stained with Hoechst 33342 Nuclear stain and observed as blue color, and autophagosomes were stained with CYTO-ID^®^ dye and observed as green color. Quantitative expression of autophagosomes in DCs was determined by the same method as above, except that the cells were only stained with the CYTO-ID^®^ dye. Next, the cells were gently trypsinized and analyzed for the percent of cells expressing autophagosomes using flow cytometry (BD Accuri C6 Plus flow cytometer; BD Bioscience, San Jose, CA, USA).

### 2.6. In Vivo Immunization Procedure and Dosing Regimen

**Figure 1:**
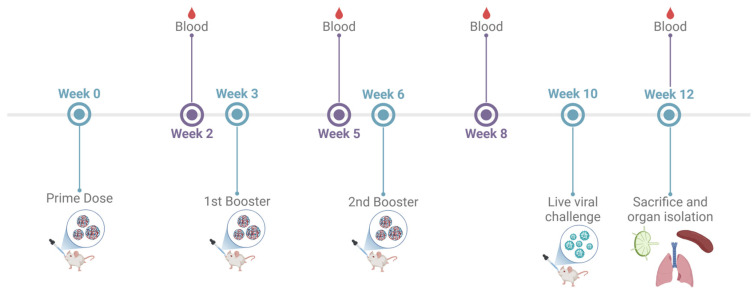
Preclinical vaccine study timeline and dosing regimen. 6–8-week-old mice were immunized with three doses at weeks 0, 3, and 6. The mice were bled 2 weeks after each dose at weeks 3, 5, 8, and 10 to evaluate serum antibody levels. The mice were challenged intranasally with 50 μL of an 0.5 × LD_50_ dose of Influenza A Virus, A/Puerto Rico/8-WG/1934 (H1N1) at week 10 and sacrificed 14 days after challenge (week 12). Terminal blood, lymph nodes, spleen, and lungs were isolated and processed for evaluation of immune responses.

The immunogenicity of the adjuvanted combination vaccine administered via the intranasal route was assessed in 6–8-week-old male Swiss Webster (CFW) mice. The testing was performed as per the approved Mercer University IACUC protocol (Animal protocol #A2411016; Approval date 20 November 2024). The antigen dose was as follows: inactivated SARS-CoV-2—20 μg/mouse; inactivated Influenza A H1N1—20 μg/mouse. The adjuvants dose was as follows: Alhydrogel^®^—30 μg/mouse; AddaVax™—5 μg/mouse; CPG 7909—20 μg/mouse. The animals were divided into three groups: No Treatment control group, IM suspension group, Adjuvanted combination IN-MP vaccine group. The animals received three doses of the vaccine at weeks 0, 3, and 6. The animals were bled 2 weeks after each dose, and the serum was collected for determining the antibody levels as described previously. The mice were challenged intranasally with 50 μL of an 0.5 × LD_50_ dose of Influenza A Virus, A/Puerto Rico/8-WG/1934 (H1N1) to assess T-cell responses of vaccinated mice compared to naïve mice post challenge. The mice were monitored for 14 days post challenge. The animals were sacrificed at week 12, and their immune organs, including the spleen and lymph node, were isolated and processed into single-cell suspensions to analyze T-cell responses (Figure 1).

### 2.7. Determining the Virus-Specific Antibody Levels in Serum and Lung Wash Samples of Vaccinated Mice

The mice were bled for two weeks after each dose, and the serum was collected to evaluate the SARS-CoV-2-specific and influenza A H1N1-specific antibody responses. An enzyme-linked immunosorbent assay was used to determine the serum IgM, IgG, IgA, IgG1 and IgG2a levels described previously [27]. For this purpose, high-binding 96-well plates (MICROLON^®^, 96 well plate, High binding, Greiner bio one purchased from Thermofisher Scientific, Waltham, MA, USA) were coated with 50 μL/well of the inactivated antigen (0.2 μg/well) (SARS-CoV-2/Influenza) in a pH 9.6 carbonate buffer solution. The coated plates were kept overnight at 4 °C to facilitate attachment of the antigen. Following incubation, plates were washed with 200 μL of 0.01% Tween-20 PBS (T-PBS) solution and blocked with 50 μL/well of 3% Bovine Serum Albumin (BSA) in T-PBS (blocking solution) for 3 h at 37 °C. The plates were rewashed, and the diluted serum sample (50 μL/well) was added to the wells and incubated overnight at 4 °C. The plates were washed again, and 50 μL/well of the HRP-tagged secondary goat anti-mouse IgM, IgG, and IgA antibodies (1:2000 to 1:4000) were added and incubated at 37 °C for 90 min. Next, the plates were washed, and 50 μL/well of the TMB (3,3′,5,5″-tetramethyl benzidine) substrate reagent (BD OptEIA™, BD Biosciences, San Jose, CA, USA) was added to each well and kept at room temperature for 10 min. The reaction was stopped by adding 50 μL of 0.3 M H_2_SO_4_ to each well. The absorbance was read immediately at 450 nm using a plate reader. The cut-off value (COV) of the measured OD was calculated as the average + 2 standard deviations (SD) of serum from Naïve (control) mice [33]. In each group, samples that had OD values above the COV was considered a positive response. 

To evaluate the IgG and IgA levels in the lung wash samples, the lungs of the mice were removed after sacrifice at week 12 and processed into single-cell suspensions as described previously [27]. The cells were centrifuged and the SARS-CoV-2-specific and Influenza A H1N1-specific IgG and IgA levels in the isolated supernatant (lung wash) was evaluated using an ELISA as described.

### 2.8. Neutralization Titers

The percent neutralization of the SARS-CoV-2 pseudovirus by the serum antibodies were evaluated using a pseudovirus neutralization assay protocol (BEI resources). Briefly, HEK 293T-hACE 2 cells were grown in a 96 well plate at a density of 1.5 × 10^4^ cells/well until they reached 70–80% confluency. The infection mix was prepared in a different 96 well round bottom plate by incubating serum samples (30 μL/well), diluted at a ratio of 1:20 in cDMEM with 20 μL/well of the pseudovirus (1:500 dilution) for 1 h at 37 °C agitated at 100 RPM. Next, the media was aspirated from the cells and 10 μL/well of polybrene transfection reagent was added at a concentration of 30 μg/mL. The cells were then inoculated with the 50 μL of the pseudovirus-antibody mix and incubated for 1–3 h at 37 °C and 5% CO_2_ after which 90 μL of D10 was added to make up the volume to 150 μL/well. The cells were incubated for 48 h at 37 °C and 5% CO_2_ following which a luciferase assay was performed to quantify the luciferase expression as per the manufacturers protocol. Briefly, the growth medium was removed from the cells and the cells were washed gently with PBS. 20 μL/well of the 1X lysis buffer was added to the wells and mixed by gentle rocking. The plate reader (synergy H1 Biotek) was programmed to perform a 2 s measurement delay followed by a 10 s measurement read for luminescence. 100 μL/well of the luciferase assay reagent and 20 μL/well of the cell lysate were added to lumitrac plates (Greiner Bio) and mixed by pipetting 2–3 times. The plate was read immediately to quantify the Relative Light Units (RLU). The cells treated with pseudovirus only (absence of antibody) was used as a positive control for luciferase expression. The percent neutralization was calculated using the following formula:

$$\%\;\mathrm{Neutralization}=\frac{\left[\left(\mathrm{RLU}\;\mathrm{of}+\mathrm{ve}\;\mathrm{control}\right)-\left(\mathrm{RLU}\;\mathrm{of}\;\mathrm{serum}\;\mathrm{sample}\right)\right]\times 100}{\left(\mathrm{RLU}\;\mathrm{of}+\mathrm{ve}\;\mathrm{control}\right)}$$

### 2.9. Evaluating the Virus-Specific T-Cell Responses in Immunized Mice

The mice were sacrificed during week 12, and spleen and lymph nodes (inguinal and brachial) were isolated and processed into single-cell suspensions as described previously [27]. The red blood cells (RBCs) in the spleen were lysed by adding ammonium chloride potassium (ACK) lysis buffer. The cells were centrifuged to remove the lysed RBCs at 1200 rpm for 10 min and the splenocytes were resuspended in fresh DMEM and stimulated with 5 μg/mL of either antigen (iSCoV-2/IIV) and 100 IU/mL IL-2 for 24 h. Lymphocytes and splenocytes contain DCs which can present the antigen to previously vaccinated antigen-primed T-cells for recall. IL-2 is a cytokine typically used to stimulate and activate antigen-primed T-cells. Thus, the antigen-specific cellular responses in the vaccinated mice were evaluated using an indirect method of stimulating the lymphocytes and splenocytes with the respective antigens and IL-2. Following 24 h stimulation, the cells were centrifuged at 1200 rpm to discard the supernatant and resuspended in 100 μL of the marker solution containing APC-labeled anti-mouse CD4 (3 µL/test) and FITC-labeled anti-mouse CD8 (3 µL/test) antibodies in PBS. The cells were incubated for 1 h on ice, protected from light, and gently vortexed every 15 min. Following incubation, the cells were washed 3 times and analyzed using BD Accuri C6 Plus Flow Cytometer (BD Bioscience, Ann Arbor, MI, USA). The no treatment control was processed and analyzed under similar conditions and the differences in the T-cell responses between the treatment groups and no treatment control was evaluated.

### 2.10. Statistical Analysis

To perform the statistical analysis, GraphPad Prism 10 software (San Diego, CA, USA) was utilized. For normally distributed data with independent groups, the one-way ANOVA test was employed. In cases of dependent groups, the two-way ANOVA test was used. To conduct multiple comparisons between two groups, a post hoc Šidák’s test was applied. For comparisons involving three or more groups, either a post hoc Tukey test for comparing means or a post hoc Dunnett test for comparing means to the control was utilized. The significance levels were defined as follows: *p* > 0.05 (ns—non-significant), *p* ≤ 0.05 (*), *p* ≤ 0.01 (**), *p* ≤ 0.001 (***), and *p* ≤ 0.0001 (****). A statistically significant result was considered for *p*-values less than 0.05. The data were presented in the format of mean ± standard error mean (SEM) or mean ± standard deviation (SD). All experiments are reported in triplicates, unless stated otherwise.

## 3. Results

### 3.1. Antigen/Adjuvant MP Characterization

The MP yield, particle size, surface charge (zeta potential), polydispersity index (PDI) of the different antigen/adjuvant MP are listed in Table 2. The yield of the formulated antigen/adjuvant MP was in the range of 88% to 92% *w*/*w*. The size of the microparticles ranged from 0.9 to 2 µm, which is ideal for recognition by antigen-presenting cells (APCs), leading to phagocytosis and subsequent antigen degradation in the cytosol [18,21]. The poly-dispersity index (PDI) ranged from 0.1 to 0.9, indicating moderate uniformity in particle size distribution. The MP had a negatively charged surface, with a zeta potential ranging from −11 mV to −20 mV. Although polyvinyl alcohol (PVA) is commonly used as a stabilizer to reduce the inherent negative charge of poly(lactic-co-glycolic acid) (PLGA), the low percentage of PVA (0.1% *w*/*v*) used in this formulation may have resulted in negatively charged particles [34]. The Alhydrogel^®^ MP had a positive surface charge of +11.01 mV which may be due to the positive charge of the entrapped Alhydrogel itself. The encapsulation efficiency of the antigen in the polymer matrix using the double emulsion method was >85% (Table 2).

### 3.2. Vaccine MP Increased NO Levels in Stimulated DCs and Were Non-Cytotoxic

**Figure 2:**
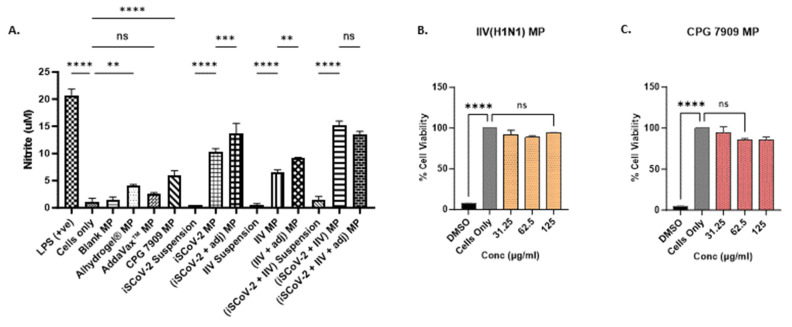
In vitro nitrite release and cytotoxicity assessment. (**A**). Nitrite released by DCs upon stimulation with various treatment groups. Cell density was adjusted to 3 × 10^4^ cells/well. The nitrite release in the supernatant was assessed using the Griess’s assay. NO plays a role in fighting invading pathogens and activation of signaling molecules in innate immunity. The MP groups produced increased levels of nitrite compared to the suspension vaccine. Addition of adjuvants potentiated the response of the MP vaccine. The (iSCoV-2 + IIV + adjuvants) MP group received only half the dose of unadjuvanted (iSCoV-2 + IIV) MP group (listed in Table 1) and produced response comparable to the unadjuvanted MP combination vaccine. Data are expressed as mean ± SEM (*n* = 3), One-way ANOVA test, Post hoc Tukey’s multiple comparisons test, ns (non-significant), ** *p* ≤ 0.01, *** *p* ≤ 0.001, and **** *p* ≤ 0.001. (**B**,**C**). Percent cell viability of DCs pulsed with varying concentrations of IIV MP (B) and CPG 7909 MP (**C**). Cell density was adjusted to 1 × 10^4^ cells/well. The cells were treated with two-fold serial dilutions of the corresponding MP groups at three concentrations, 31.25 µg/mL, 62.5 µg/mL, 125 µg/mL in cDMEM (100 μL/well) for 24 h. 25% *v*/*v* DMSO was used as a -ve control, and cells only were used as a +ve control. The % cell viability was >95% for all concentrations tested. Data are expressed as mean ± SEM (*n* = 3). One-way ANOVA test, Post hoc Dunnett’s multiple comparison test, **** *p* ≤ 0.0001, ns, non-significant.

The release of nitric oxide (NO) by the dendritic cells (DCs) pulsed with vaccine microparticles was measured and quantified as an indicator of innate immune response activation [35]. NO plays a significant role in combating invading pathogens and serves as an important signaling molecule in innate immunity [29]. The production of NO by the stimulated DCs was quantified using a Griess Nitrite Release Assay, showing that the microparticle vaccine groups produced significantly higher levels of NO compared to the corresponding suspension groups, indicating that the microparticle encapsulation of the vaccine antigen can enhance the immune recognition of the antigen (Figure 2A). Furthermore, the addition of adjuvant microparticles potentiated the response of the single-antigen vaccine microparticles. The individual adjuvant MPs were also evaluated for NO release. The cells treated with Alhydrogel^®^ MP and CPG 7909 MP resulted in significant NO levels. No significant NO response was observed for the Addavax™ MP. These data suggest that encapsulating the adjuvants in polymer matrices increases the recognition of the cargo due to its larger size and triggers the release of NO. Thus, to assess the combined vaccine antigen microparticles (SARS-CoV-2 + Influenza) with adjuvants, the antigen dose was reduced to half of the original concentration (see Table 1). The results indicated that the addition of adjuvants to the combined vaccine microparticles produced NO levels similar to the unadjuvanted combined vaccine microparticle group, allowing for the reduction in the antigen dose used for in vivo immunization (Figure 2A). 

The vaccine microparticles must be capable of eliciting an immune response without being cytotoxic to the target cells. The cytotoxicity of the iSCoV-2 MP, Alhydrogel^®^ MP, and AddaVax™ MP, had been previously tested and reported [27]. Here, the cytotoxicity of the IIV MP and the CPG 7909 microparticles when exposed to DCs was evaluated using three different doses with a 2-fold increase, employing an MTT assay as described previously. The vaccine microparticles and adjuvant microparticles were found to be non-cytotoxic and resulted in no significant cell death up to a concentration of 125 μg/mL. A 25% *v*/*v* Dimethyl sulfoxide (DMSO) was used as a positive control and resulted in a significant decrease in the percentage of cell viability compared to the cells-only control (Figure 2B,C).

### 3.3. Vaccine MP Promotes Autophagy in DCs

**Figure 3:**
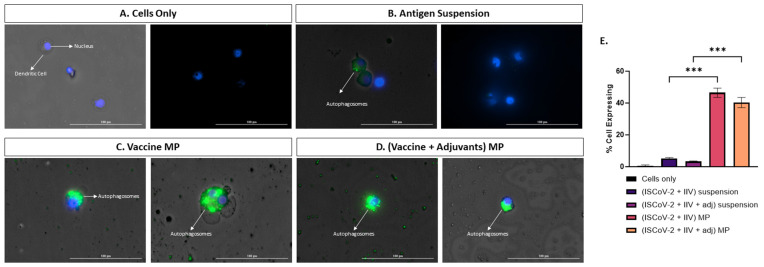
Assessing autophagy in DCs treated with different groups A-D. Fluorescent microscope imaging. (**A**). Cells only (−ve control). (**B**). Antigen suspension. (**C**). Vaccine MP. (**D**). Vaccine + Adjuvants in MP. (**E**). Flow cytometry analysis of autophagy. The vaccine MP induces autophagy in DCs which is significantly higher than the antigen suspension group (**E**). The anitgen suspension induces autophagy to a lesser extent (**B**). Data expressed as mean ± SEM, n = 4, one-way ANOVA, post hoc Tukey’s multiple comparisons test. *** *p* ≤ 0.001.

Autophagosome formation plays a crucial role in the activation, maturation, cytokine release, antigen presentation, and T-cell activation of dendritic cells (DCs) upon stimulation [20]. Autophagy is closely associated with the cross-presentation of extracellular antigens via the MHC I pathway and the activation of CD8 T-cell response, which is essential in combatting viral antigens [19]. In this study, we assessed the capacity of the MP vaccine to induce autophagy in stimulated DCs. The percentage of cells expressing autophagy was higher in the cells treated with the adjuvanted MP vaccine compared to its corresponding suspension form, indicating that the MP carrier system enhances autophagosome formation (Figure 3E). Visual images in Figure 3A–D demonstrate higher levels of autophagosome formation in the MP vaccine group compared to other test groups.

### 3.4. Virus-Specific Antibody Levels in Serum and Lung Wash Samples and Percent Neutralization of SARS-CoV-2

**Figure 4:**
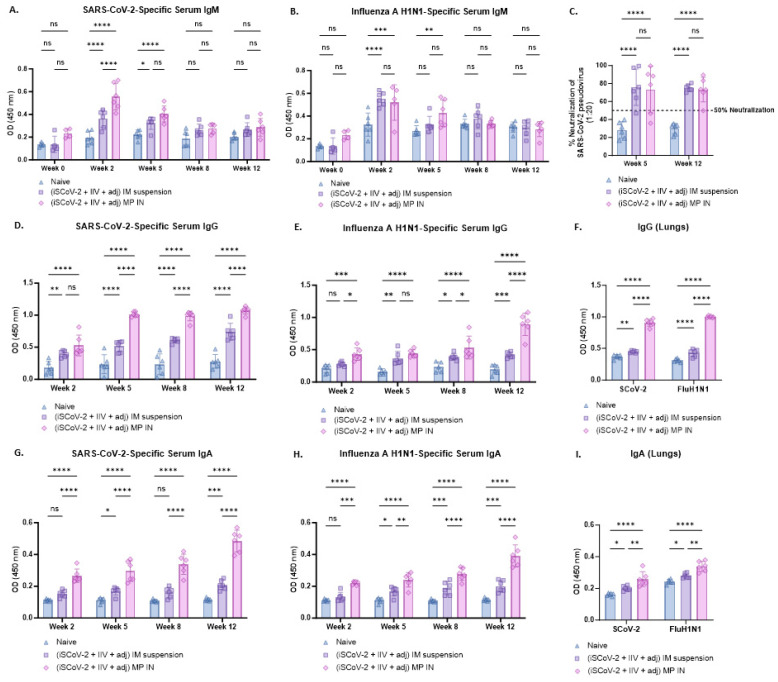
Virus-specific antibody levels in the serum and lung wash of vaccinated mice. The serum antibodies were assessed for their ability to bind to inactivated SARS-CoV-2 or inactivated influenza A H1N1 using ELISA. The functional activity of the serum antibodies was assessed for SARS-CoV-2 using a pseudovirus neutralization assay. IgG and IgA levels in the lung wash of the vaccination mice were assessed at week 12 using ELISA. (**A**). SARS-CoV-2-specific IgM. (**B**). Influenza A H1N1-specific IgM. Serum IgM levels in vaccinated mice peaked at week 2 after the prime dose and subsequently decreased in the following weeks. (**C**). % neutralization of SARS-CoV-2 pseudovirus by serum antibodies was assessed at weeks 5 and 12. The neutralizing capacity of the antibodies varied for each animal and was found to range between 47 and 99% at week 5, and between 50% and 90% at week 12 for the IN-MP vaccine group. (**D**). SARS-CoV-2-specific IgG. (**E**). Influenza A H1N1-specific IgG. The serum IgG levels increased after the prime dose and remained high until week 12. The IN-MP vaccine group exhibited higher antibody levels compared to the IM suspension group. SARS-CoV-2-specific antibody levels dominated over the influenza-specific antibody levels. (**F**). Virus-specific IgG in lung wash samples. The IN-MP vaccine induced significant IgG levels in lungs. (**G**). SARS-CoV-2-specific IgA. (**H**). Influenza A H1N1-specific IgA. The serum IgA levels of the IN-MP vaccine were higher than the IM suspension vaccine for both viruses. (**I**). Virus-specific IgA in lung wash samples. The IN-MP vaccine induced significant IgA responses in the lung wash samples of vaccinated mice. Responses obtained are compared to no treatment (control) and IM suspension (control) group. Data are expressed as individual values, n = 6. Two-way ANOVA, post hoc Tukey’s multiple comparisons test. ns, non-significant, * *p* ≤ 0.05, ** *p* ≤ 0.01, *** *p* ≤ 0.001, **** *p* ≤ 0.0001.

The serum antibody levels following immunization were evaluated 2 weeks after each dose. The virus-specific IgM, IgG, and IgA antibody levels were identified and measured using ELISA. In both the IN-MP vaccine and the IM suspension vaccine groups, the serum IgM levels reached their peak during week 2 following the initial dose and then decreased in the subsequent weeks, possibly due to a switch from IgM to IgG/IgA isotypes [36]. The IN-MP vaccine resulted in significantly higher levels of SARS-CoV-2-specific IgM at week 2 compared to the IM suspension vaccine, while no significant differences were observed between the two routes for the Influenza-specific IgM levels (Figure 4A,B). 

Following the prime dose at week 2, the IN-MP vaccine induced notably high levels of virus-specific serum IgG, which remained elevated up to week 12. The IM suspension vaccine also elicited significant virus-specific serum IgG responses, but the IN-MP group exhibited significantly higher responses than the IM suspension group. The SARS-CoV-2-specific serum IgG levels appeared to predominate over the Influenza-specific IgG response, although the significance between the two virus-specific serum IgG levels was not assessed. It is important to note that the Influenza-specific IgG levels increased at week 12 after the intranasal challenge with live Influenza virus at week 10 (Figure 4D,E). Further, the functional response of the serum antibodies was evaluated by testing their ability to neutralize the SARS-CoV-2 pseudovirus. The neutralizing capacity of the antibodies varied for each animal and was found to range between 47 and 99% at week 5, and between 50% and 90% at week 12 for the IN-MP vaccine group. Similar responses were obtained for the group that received IM suspension vaccine (Figure 4C). 

The virus-specific serum IgA levels of the group that received the IN-MP vaccine significantly increased after the prime dose at week 2 and remained elevated up to week 12. In contrast, the group that received the IM suspension vaccine produced significant serum IgA responses only after the first booster dose. The virus-specific serum IgA levels of the mice that received the IN-MP vaccine were significantly higher compared to the IM suspension group (Figure 4G,H). 

The IgG and IgA levels in the lung wash samples at week 12 were assessed using ELISA, and both routes of vaccine administration elicited IgG and IgA responses in vaccinated mice. The IN-MP vaccine induced significantly higher levels of IgG and IgA in the lungs of the vaccinated mice compared to the IM suspension vaccine (Figure 4F,I).

**Figure 5:**
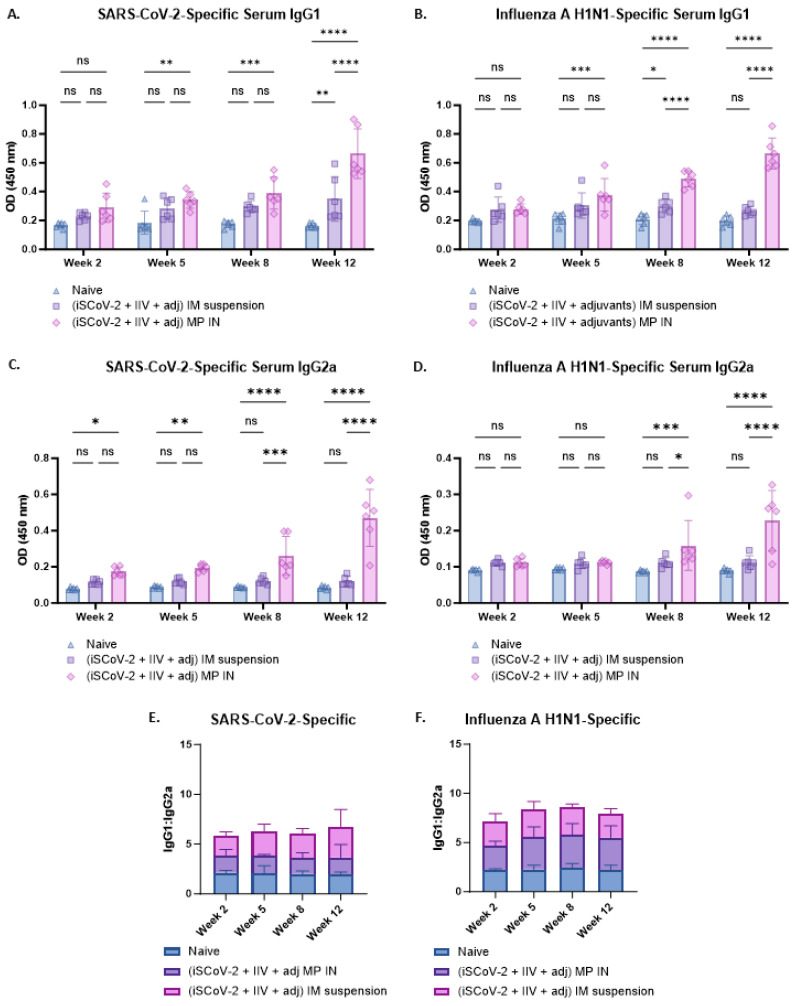
Virus-specific IgG1 and IgG2a levels and IgG1/IgG2a ratio in the serum of vaccinated mice. (**A**). SARS-CoV-2-specific IgG1. (**B**). Influenza A H1N1-specific IgG1. (**C**). SARS-CoV-2-specific IgG2a. (**D**). Influenza A H1N1-specific IgG2a. (**E**). SARS-CoV-2-specific IgG1/IgG2a. The SARS-CoV-2-specific IgG1/IgG2a ratio was evaluated which indicated that the mean value was 3.6 for the IN-MP vaccine and 6.2 for IM suspension vaccine. (**F**). Influenza A H1N1-specific IgG1/IgG2a. For influenza, the mean IgG1/IgG2a was 5.4 for the IN-MP vaccine and 7.9 for the IM suspension vaccine. The Responses obtained are compared to no treatment (control) and IM suspension (control) group. Data are expressed as individual values, n = 6. Two-way ANOVA, post hoc Tukey’s multiple comparisons test. ns, non-significant, * *p* ≤ 0.05, ** *p* ≤ 0.01, *** *p* ≤ 0.001, **** *p* ≤ 0.0001.

The antibody responses were further characterized by evaluating the IgG1 and IgG2a levels using ELISA (Figure 5A–D). A Th-1 biased response is characterized by increased IgG2a levels whereas a Th-2 biased response is characterized by increased IgG1 levels [37]. A IgG1/IgG2a ratio of ≤0.5 indicates increased IgG2a levels and Th-1 biased, predominantly cell-mediated responses, which is a characteristic of intracellular pathogens and viruses [37]. A IgG1/IgG2a ratio between 0.5 and 2.0 suggests mixed Th-1 and Th-2 responses and an IgG1/IgG2a ratio ≥ 2.0 is indicative of increased IgG1 levels and suggests a Th-2 biased, humoral response [37,38]. The SARS-CoV-2-specific IgG1/IgG2a ratio was assessed from the IgG1 and IgG2a levels (Figure 5A,C). The mean value was 3.6 for the IN-MP vaccine and 6.2 for IM suspension vaccine. The individual IgG/IgG2a values for the IN-MP vaccine were between 0.8 and 3.5, with more data points with a ratio below 2.0 indicating a predominantly mixed Th-1 and Th-2 response, whereas for the IM suspension vaccine, more data points were at a ratio > 2.0, indicating a strong Th-2 biased (humoral) response (Figure 5E). The IgG1/IgG2a ratio was also evaluated for the influenza A H1N1-specific antibodies from the IgG1 and IgG2a levels (Figure 5B,D). For influenza, the mean IgG1/IgG2a ratio was 5.4 for the IN-MP vaccine and 7.9 for the IM suspension vaccine. For both routes of administration, the influenza-specific individual data points were strongly Th-2 biased (humoral) (Figure 5F).

### 3.5. Virus-Specific T-Cell Responses in Lymphocytes and Splenocytes of Vaccinated Mice

**Figure 6:**
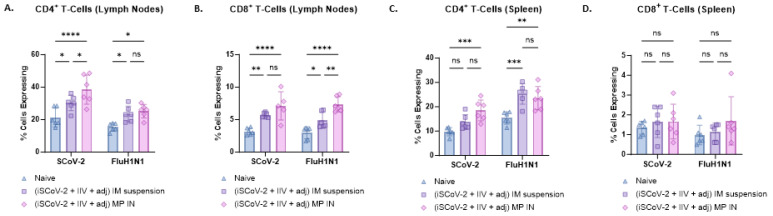
SARS-CoV-2-specific and Influenza A H1N1-specific CD4+ and CD8+ T-cells in the splenocytes and lymphocytes of vaccinated mice. The antigen-specific responses were evaluated by indirectly stimulating the cells with the respective antigen (5 μg/mL) and IL-2 (100 IU/mL) to activate antigen-primed T-cells in vaccinated mice. The % cells expressing CD4 and CD8 on the T-cell surface was quantified using flow cytometry. The IN-MP vaccine induced high percentages of antigen-specific T-cells expressing CD4 and CD8 levels in the lymphocytes and only CD4+ T cells in the spleen. (**A**). CD4+ T-cells in the lymphocytes. (**B**). CD8+ T-cells in the lymphocytes. (**C**). CD4+ T-cells in the splenocytes (**D**). CD8+ T-cells in the splenocytes. Responses obtained are compared to no treatment (control) and the IM suspension (control) group. Data are expressed as individual values, n = 6, Two-way ANOVA, post hoc Tukey’s multiple comparisons test. ns, non-significant, * *p* ≤ 0.05, ** *p* ≤ 0.01, *** *p* ≤ 0.001, **** *p* ≤ 0.0001.

Flow cytometry analysis was used to evaluate CD4+ and CD8+ T-cells in the lymphocytes and splenocytes of vaccinated mice. Antigen-specific responses were assessed by stimulating the cells with the respective antigen and IL-2 to activate antigen-primed T-cells in vaccinated mice. The IN-MP vaccine led to significant antigen-specific CD4+ T-cell responses in the lymphocytes and splenocytes of vaccinated mice compared to the control group with no treatment. The percentage of SCoV-2-specific CD4+ T-cells in the lymphocytes was higher than the percentage of influenza-specific CD4+ T-cells. The IN-MP vaccine induced CD4+ T-cell responses that were comparable or higher than those of the IM suspension vaccine (Figure 6A,C). Significant antigen-specific CD8+ T-cell responses were also observed in the lymphocytes of mice vaccinated with the IN-MP vaccine, with no differences observed in the splenocytes. The CD8+ T-cell response of the IN-MP vaccine was comparable or higher than that of the IM suspension vaccine. The percentage of antigen-specific CD8+ T-cells in the lymphocytes for SCoV-2 and influenza were found to be similar (Figure 6B,D).

## 4. Discussion

The study aimed to develop an inactivated polymeric combination vaccine for SARS-CoV-2 and influenza and evaluate its ability to generate immune responses in mice when administered via the IN route. In vitro, the vaccine MP triggered an innate immune response and did not show any toxicity to cells. In vivo, the vaccine induced higher serum antibody levels compared to the IM suspension vaccine, with neutralizing activity against SARS-CoV-2 pseudovirus observed in the serum antibodies. Additionally, IgG and IgA were detected in the lung wash samples, and the IN-MP vaccine also induced antigen-specific T-cell responses in the lymphocytes and splenocytes of the vaccinated mice.

The successful development of an intranasal vaccine depends on factors such as antigen selection, adjuvants, and vaccine formulation strategy [39]. The nasal mucosa, consisting of thick mucus and cilia, serves as a protective barrier against pathogens, and vaccine formulation strategy is crucial for the residence time of the antigen in the nasal mucosa [10,11,12]. In this study, PLGA was utilized to encapsulate vaccine antigens and adjuvants, aiming to protect the cargo and improve sustained delivery [21,40,41]. Previous studies with PLGA have shown sustained release properties, which can aid in increased residence time of the vaccine antigen in the nasal mucosa [40]. The size of the vaccine MP, around 0.9–2 μm, plays a pivotal role in its recognition by the APC to facilitate phagocytosis [18,42]. Furthermore, evidence of autophagy formation in DC when treated with MP suggests the potential cross-presentation of the antigen via the MHC I pathway and subsequent activation of CD8+ T-cells, supported by the increased antigen-specific T-cell responses observed in the vaccinated mice [19,20,32]. It was essential to ensure that the vaccine MP formulation was non-cytotoxic to target cells while maintaining the immunogenic properties of the antigen/adjuvant prior to in vivo testing. The vaccine MP generated an innate immune response evaluated by the release of NO, a molecule involved in the activation of downstream signaling pathways of immune activation, and was also non-cytotoxic at concentrations as high as 125 µg/mL [29,35].

The vaccine was administered to mice in three doses: one prime and two boosts, each 21 days apart, in vivo. The serum antibody levels were evaluated at day 14 after each dose. After the prime dose, the serum IgM levels peaked at day 14 and gradually decreased in the later weeks. IgM, a multivalent antibody, is the first line of defense against invading pathogens and is typically observed early in the humoral immune response [36]. IgM provides fast protective immunity against viral infections and undergoes isotype class switching to IgG with increased antigen affinity [36]. Following the prime dose with the MP vaccine, the serum IgG levels increased and remained elevated until week 12. The IgG specific to SARS-CoV-2 antigen seemed to dominate over the influenza-specific IgG level based on absorbance values, but further investigation is required to characterize the dominance of one antigen over another in a combination vaccine. At week 10, the mice were intranasally challenged with a sublethal dose of the influenza A H1N1 strain, which boosted the IgG levels at week 12. Similarly to IgG, serum IgA levels increased after the prime dose and remained elevated until week 12. IgA, along with IgG, is capable of binding to the viral pathogen and facilitating neutralization and pathogen killing. The MP vaccine elicited antigen-specific IgG and IgA responses. Furthermore, the virus-specific IgG and IgA in lung wash following vaccination with the IN-MP vaccine suggest the presence of localized antibodies in the lungs.

The functional response of the antibodies to prevent SARS-CoV-2 viral entry into the target cells was evaluated using a pseudovirus neutralization assay. The neutralizing capacity of the antibodies varied for each animal, ranging between 47% and 99% at week 5, and between 50% and 90% at week 12 for the IN-MP vaccine group. The variation in neutralization capacity could be due to factors such as antigen type, adjuvants, and administration technique of the IN vaccine, as well as variation in dose. Stronger neutralization can be achieved by utilizing different antigen types, such as mRNA or subunit proteins. The currently marketed intranasal flu vaccine, Flumist^®^ (AstraZeneca, Cambridge, UK), employs a live-attenuated form of the influenza virus to generate a strong immune response [43]. However, live attenuated vaccines are not as safe and tolerable as inactivated or subunit vaccines [43]. Different antigen types and adjuvants can be tested using the IN-MP vaccine delivery system to enhance immune responses. Additionally, using an IN device to regulate the dose of antigen during vaccine administration can minimize variation in individual responses. 

Further characterization of the IgG subtypes, IgG1 and IgG2a and the IgG1/IgG2a ratio revealed that the responses specific to SARS-CoV-2 were mixed and suggests the activation of both Th-1 and Th-2 mediated responses. However, the Influenza-specific responses were predominantly Th-2 biased. A Th-1-biased response is characterized by cellular responses and increased IgG2a levels whereas a Th-2-biased response is characterized by humoral responses and increased IgG1 levels [37,38]. A vaccine is considered efficacious if it can induce both humoral (antibody-mediated) and cellular (B-cell and T-cell) mediated immunity [11,12]. The ability of the IN-MP vaccine in inducing T-cell responses was evaluated by assessing CD4 and CD8 T-cell surface marker expression, which showed an increase in the percentage of cells expressing T-cell markers upon vaccination and subsequent restimulation. CD4+ cells play a role in activating B-cells to produce antibodies, while CD8+ T-cells can function by directly killing pathogen-infected cells. The data summarized in this manuscript suggest the presence of both humoral and cellular immune responses. This may be due to many factors such as the internal trafficking of PLGA MP vaccine, degradation pathways, and adjuvants used in the study. However, cytokine profiling and further T-cell phenotype evaluation is required to draw definitive conclusions of the Th-bias in response.

The evaluation of the IN-MP vaccine was conducted in the context of a preliminary proof-of-concept, and certain limitations were identified which will be addressed in future work. To properly mimic the marketed vaccine for flu and COVID, the proposed vaccine needs to be tested with a trivalent flu antigen and the updated variant for the SARS-CoV-2. In future studies, responses from the investigational vaccine and the marketed vaccine will be compared, including the approved Flumist vaccine, and other approved COVID and influenza vaccines. In the context of immune response generation, the vaccine will be further evaluated by including lethal challenges for both viruses, assessing the neutralizing activity of antigen-specific antibodies, conducting tetramer pull-down of antigen-specific T-cells and B-cells, testing the functional activity, and conducting immunophenotyping to examine T-cell and B-cell memory populations. Additionally, the IN administration of the vaccine can induce localized responses at the upper and lower respiratory tract, which will also be evaluated.

## 5. Conclusions

This proof-of-concept study provides a summary of the preliminary immunogenicity assessment findings for an inactivated polymeric combination vaccine designed for COVID-19 and influenza, which was tested using the intranasal (IN) route of administration. The study is supported by data that prove the vaccine’s capability to elicit immune responses both in vitro and in vivo. Moreover, the findings indicate that the polymeric combination vaccine, referred to as the IN-MP vaccine, generated immune responses that were comparable to or even higher than those produced by the intramuscular (IM) suspension vaccine. As a result, this suggests that the IN-MP vaccine could be further investigated as a promising alternative to IM injections.

## Figures and Tables

**Table 1:** Description of treatment groups and dose for the in vitro studies.

Treatment Groups	Description and Dose
No Treatment (−ve control)	Cells only
LPS (+ve control)	lipopolysaccharide (LPS) from *Escherichia coli* (2 μg/mL)
Blank MP	N/A
Alhydrogel^®^ MP	3 μg/mL
AddaVax™ MP	0.5 μg/mL
CPG 7909 MP	2 μg/mL
iSCoV-2 Suspension	Inactivated SARS-CoV-2 (iSCoV-2) antigen suspension (2 μg/mL)
IIV Suspension	Inactivated Influenza Virus (IIV) A H1N1 antigen suspension (2 μg/mL)
(iSCoV-2 + IIV) Suspension	Inactivated SARS-CoV-2 (2 μg); Inactivated Influenza A H1N1 (2 μg/mL) antigen suspension
iSCoV-2 MP	Inactivated SARS-CoV-2 microparticles (2 μg/mL)
IIV MP	Inactivated Influenza A H1N1 microparticles (2 μg/mL)
(iSCoV-2 + IIV) MP	Inactivated SARS-CoV-2 (2 μg/mL); Inactivated Influenza A H1N1 (2 μg/mL) microparticles
(iSCoV-2 + adjuvants) MP	Inactivated SARS-CoV-2 (2 μg/mL); Alhydrogel^®^MP (3 μg/mL); AddaVax™ MP (0.5 μg/mL); CPG 7909 (2 μg/mL) microparticles
(IIV + adjuvants) MP	Inactivated Influenza A H1N1 (2 μg/mL); Alhydrogel^®^MP (3 μg/mL); AddaVax™ MP (0.5 μg/mL); CPG 7909 (2 μg/mL) microparticles
(iSCoV-2 + iFlu + adjuvants) MP	Inactivated SARS-CoV-2 (1 μg/mL); Inactivated Influenza A H1N1 (1 μg/mL); Alhydrogel^®^MP (3 μg/mL); AddaVax™ MP (0.5 μg/mL); CPG 7909 (2 μg/mL) microparticles

**Table 2:** Characterization of Vaccine/Adjuvant Microparticles.

S.No	Parameter	Mean ± Standard Deviation (SD)				
		Inactivated SARS-CoV-2 MP	Inactivated Influenza A H1N1 MP	Alhydrogel^®^ MP	AddaVax™ MP	CPG 7909 MP
1.	Product yield (%)	89 ± 5	87.5 ± 5	88.5 ± 5	91.5 ± 5	90.8 ± 5
2.	Particle size (nm)	950 ± 153.4	998 ± 50.1	1432 ± 278.6	1396 ± 259.1	1100 ± 123.4
3.	Zeta potential (mV)	−21.04 ± 1.41	−22.06 ± 2.03	+11.01 ± 0.252	−13.05 ± 2.65	−19.06 ± 1.99
4.	PDI	0.55 ± 0.048	0.17 ± 0.033	0.954 ± 0.04	0.896 ± 0.069	0.47 ± 0.045
5	% EE	85.4%	88.5%	-	-	-

## Data Availability

Data will be made available on reasonable request.
